# Treatment of Cervical Chlamydial Infection With Amoxicillin/Clavulanate Potassium

**DOI:** 10.1155/S1064744993000249

**Published:** 1993

**Authors:** Melinda S. Mann, Sebastian Faro, Maurizio L. Maccato, Raymond H. Kaufman

**Affiliations:** 1 Private practice Brooklyn NY USA; 2 Department of Gynecology and Obstetrics University of Kansas School of Medicine 3901 Rainbow Blvd Kansas City KS 66160-7316 USA; 3 Department of Obstetrics and Gynecology Baylor College of Medicine Houston TX USA

## Abstract

*Objective:* To determine if amoxicillin/clavulanate potassium is effective in the treatment of *Chlamydia trachomatis* endocervicitis.

*Methods:* Thirty-two patients with culture-proven endocervical infection were treated with amoxicillin/clavulanate potassium, 500 mg orally 3 times a day for 10 days. Post-treatment endocervical specimens were obtained at 2, 4, and 6 weeks for culture of *C. trachomatis*. Male partners were treated with doxycycline, 100 mg orally twice daily for 10 days. The couples were provided condoms and asked to use them throughout the duration of the study.

*Results:* All patients treated with amoxicillin/clavulanate potassium were cured of signs of cervicitis. All were found to be free of *C. trachomatis* at their follow-up visits.

*Conclusions:*  Amoxicillin/clavulanate potassium is effective in eradicating *C. trachomatis.*


Infectious Diseases in Obstetrics and Gynecology 1:104–107 (1993)
© 1993 Wiley-Liss, Inc.

### Treatment of Cervical Chlamydial Infection With Amoxicillin/Clavulanate Potassium

**Melinda S. Mann, Sebastian Faro, Maurizio L. Maccato, and Raymond H. Kaufman**

*Private practice, Brooklyn, NY (M.S.M.), Department of Gynecology and Obstetrics, University of Kansas School of Medicine, Kansas City, KS (S.F.), and Department of Obstetrics and Gynecology, Baylor College of Medicine, Houston, TX (M.L.M., R.H.K.)*

ABSTRACT

***Objective:*** **To determine if amoxicillin/clavulanate potassium is effective in the treatment of *Chlamydia trachomatis* endocervicitis.**

***Methods:*** **Thirty-two patients with culture-proven endocervical infection were treated with amoxicillin/clavulanate potassium, 500 mg orally 3 times a day for 10 days. Post-treatment endocervical specimens were obtained at 2, 4, and 6 weeks for culture of *C. trachomatis.* Male partners were treated with doxycycline, 100 mg orally twice daily for 10 days. The couples were provided condoms and asked to use them throughout the duration of the study.**

***Results:*** **All patients treated with amoxicillin/clavulanate potassium were cured of signs of cervicitis. All were found to be free of *C. trachomatis* at their follow-up visits.**

***Conclusions:*** **Amoxicillin/clavulanate potassium is effective in eradicating *C. trachomatis.***
© 1993 Wiley-Liss, Inc.

KEY WORDS
Penicillin-class, cervicitis, bacteria

*Chlamydia trachomatis* is considered to be the most common sexually transmitted bacterium in the United States. It is estimated that 4 million infections occur per year.[1] In the female, *C. trachomatis* is one etiologic agent responsible for cervicitis, urethritis, endometritis, and salpingitis.[2–5] *C. trachomatis* has been associated with abortion, premature rupture of amniotic membranes, premature labor, chorioamnionitis, and postpartum endometritis.[6–10] Like other sexually transmitted diseases, *C. trachomatis* can be found in association with other bacteria, e.g., *Neisseria gonorrhoeae* in 60% of cases.[11] Standard treatment has been tetracycline in the non-pregnant patient and doxycycline or erythromycin in the pregnant patient. Recently, clindamycin has been shown to be effective and synergistic when combined with gentamicin.[12] Various penicillins have been shown to be effective in vitro and in vivo.[13–16]

The present study was undertaken to determine if the combination of amoxicillin plus clavulanate potassium (Augmentin, Beecham Laboratories, Bristol, TN) is effective in eradicating *C. trachomatis.* This agent was chosen for its broad spectrum of activity. In vitro studies have shown that it is effective against *Gardnerella vaginalis* (S. Faro, personal communication), a significant advantage because patients with *C. trachomatis* are commonly found to be positive for *G. vaginalis* as well. In addition, the presence of *G. vaginalis* may indicate an abnormal vaginal flora dominated by anaerobic bacteria, e.g., bacterial vaginosis. Augmentin is

**Address correspondence/reprint requests to Dr. Sebastian Faro, Department of Gynecology and Obstetrics, University of Kansas School of Medicine, 3901 Rainbow Blvd., Kansas City, KS 66160-7316.**

Received June 16, 1993
Accepted August 12, 1993

suitable not only against anaerobes but also against facultative anaerobes and gram-positive aerobes. Augmentin has also been shown to be effective against β-lactamase-producing *N. gonorrhoeae*. For these reasons, it has the potential for being suitable as a single agent for the treatment of *C. trachomatis*, *N. gonorrhoeae*, and bacterial vaginosis.

### SUBJECTS AND METHODS

Study subjects were chosen from the patients seen at the colposcopy clinic of the Department of Obstetrics and Gynecology at Baylor College of Medicine. Patients 18 years of age or older were enrolled if they had chlamydia-positive endocervical cultures, had not used antibiotics within the previous 30 days, and had given written informed consent. If otherwise eligible, patients younger than 18 but older than 13 years of age were enrolled if written informed consent and parental written consent had been obtained. Patients who were allergic to or suspected of being allergic to penicillin or who admitted to having more than one sexual partner were excluded from the study.

The male partners of all patients enrolled in the study were treated with doxycycline, 100 mg orally twice daily for 10 days. An ample supply of condoms was given to each patient for the entire duration of the study. Patients who failed to use the condoms during sexual intercourse were dropped from the study. All female patients enrolled in the study were treated with Augmentin, 500 mg orally 3 times a day for 10 days. Patients were instructed not to take any other antibiotics during the study period. Patients were asked to return at 2, 4, and 6 weeks after completion of therapy for test-of-cure cultures.

Specimens for the isolation and culture of *C. trachomatis* were obtained from the endocervix using a sterile Dacron swab mounted on a plastic shaft. The portio of the cervix was first cleansed by gently wiping away with a large cotton swab any vaginal discharge that may have been present. The sterile Dacron swab was placed deep into the endocervical canal and rotated in a clockwise manner for 15 to 30 sec. The swab was then placed into Bartel's transport medium (Bartel, Bellevue, WA) and taken immediately to the laboratory for processing.[17] *C. trachomatis* was isolated on cycloheximide-treated McCoy cells (Viromed Laboratories, Minnetonka, MN). *C. trachomatis* inclusions were identified by staining with monoclonal immunoflurorescent antibody (Ortho Diagnostics, Raritan, NJ). All specimens were passed blindly twice in McCoy tissue cultures.

**TABLE 1. Results of treating *Chlamydia trachomatis* with Augmentin**

| No. of patients | No. of weeks | Culture results |
|---|---|---|
| 4 | 0 | Not done |
| 5 | 2 | Negative |
| 4 | 2, 4 | Negative |
| 18 | 2, 4, 6 | Negative |

### RESULTS

Thirty-five patients were enrolled in the study and 32 (91%) completed the study by having at least one follow-up post-treatment culture. Three patients were excluded from the study: 1) one patient became dizzy while taking Augmentin and voluntarily discontinued the medication; 2) one was seen by another physician and given doxycycline; and 3) one failed to use condoms.

Nineteen patients were black (59%), nine were Hispanic (29%), and four were Caucasian (12.5%). The ages ranged from 14 to 66 years old, with a mean of 24 years. The population may reflect the fact that the colposcopy clinic serves primarily an indigent population made up largely of black and Hispanic patients.

Augmentin was well tolerated, with minimal adverse effects. Two patients developed nausea and vomiting. However, when they took the medication after meals, they had no further problems and completed a full course of therapy. Two patients developed vaginal discharge and itching. Examination at their initial follow-up evaluations revealed the presence of fungal pseudohyphae, and a diagnosis of *Candida* vaginitis was made. Both patients were treated successfully with butoconazole.

Thirty-two patients completed a 10-day course of Augmentin. Nine patients returned only for their initial 2-week visit. Five patients returned for their 2- and 4-week visits, and 18 patients returned for their 2-, 4-, and 6-week visits (Table 1).

Thirty-one patients had negative follow-up cultures of the endocervix. However, one patient found to be negative at the 2-week visit was positive at the 4-week visit. She admitted to having had unprotected intercourse after the 2-week post-therapy evaluation. She was re-treated with Augmentin and subsequently found to be negative at the next post-therapy evaluation.

One 66-year-old patient was particularly interesting. She had been a widow for 11 years and stated that she had not engaged in sexual activity since her husband died. She was found to harbor *C. trachomatis* in her endocervical canal. This patient's culture was strongly positive, that is, there were numerous inclusion bodies present. Therefore, the possibility of it being a false-positive culture could be unlikely. Augmentin was successful in eradicating *C. trachomatis,* as she was found to have negative cultures at 2, 4, and 6 weeks post-therapy.

### DISCUSSION

The current recommended therapy for cervical chlamydial infection in the non-pregnant patient is doxycycline, 100 mg orally twice daily for 7 to 10 days. A main disadvantage of doxycycline is its relatively narrow spectrum of antimicrobial activity. Thus, it is frequently prescribed as an "add-on" antibiotic for patients in whom pelvic inflammatory disease is suspected or diagnosed.

Chlamydial infection in the pregnant patient is primarily treated with erythromycin, 500 mg orally 4 times a day for 7 to 10 days. However, erythromycin frequently causes gastrointestinal disturbances, which, in pregnancy, represent a common complication. Therefore, in the pregnant patient treated with erythromycin, compliance is a significant problem.

Azithromycin, a new macrolide, is approved for the treatment of *C. trachomatis* in a single daily dose. However, this agent has not been approved for use in pregnancy nor in the treatment of gonorrhea. Ofloxacin has been shown to be effective and has been approved for the treatment of both *N. gonorrhoeae* and *C. trachomatis*. However, quinolones are contraindicated in pregnant patients, breast feeding patients, and children under the age of 16. In addition, ofloxacin has a limited spectrum of activity against anaerobes; therefore, its use in those patients with concomitant bacterial vaginosis would be questionable.

Hospitalized patients with acute salpingitis are currently treated with cefoxitin, ceftizoxime, cefotetan plus doxycycline, or clindamycin plus gentamicin. Ambulatory treatment of pelvic inflammatory disease mainly relies on ceftriaxone plus doxycycline. Ceftriaxone does not provide adequate coverage against anaerobes, and one must question the logic of using a single dose of ceftriaxone, although it has a long half-life, to prevent progressive salpingitis. The goal of treating acute salpingitis is to prevent damage to the fallopian tube, thereby reducing the risk of ectopic pregnancy and/or infertility. Despite the reported side effect of diarrhea, which might limit its use,[5] Augmentin would seem a more logical choice in treating the ambulatory patient because of its broader spectrum of activity.

Recently, amoxicillin has been shown to have moderate in vitro anti-chlamydial activity. Clinical trials with amoxicillin used in different dosing regimens have demonstrated efficacy in treating chlamydial infection.[8,16] Other penicillins have been shown to have anti-chlamydial activity, namely, mezlocillin, ticarcillin, timentin, and piperacillin.[14–17]

Augmentin was chosen for study because it is active against penicillinase-producing strains of *N. gonorrhoeae, G. vaginalis,* and gram-positive organisms, e.g., *Streptococcus agalactiae, Staphylococcus aureaus,* and *Enterococcus faecalis*. Augmentin is active against many members of the Enterobacteriaceae and anaerobes. The presence of clavulanate potassium, a β-lactamase inhibitor, increases the spectrum of activity of amoxicillin, especially against β-lactamase-producing bacteria. Our study showed that this antibiotic is effective therapy for *C. trachomatis* infections.

Augmentin presents the opportunity of using a single agent for the treatment of *N. gonorrhoeae* and *C. trachomatis* cervicitis. Augmentin also affords the opportunity to treat bacterial vaginitis in the non-pregnant and pregnant patient who is not allergic to penicillin. Additional studies are needed to confirm its activity in the treatment of bacterial vaginosis. Its spectrum of activity makes it a potential agent for the treatment of pelvic inflammatory disease in an ambulatory setting.

Although the activity of Augmentin is probably no better than ampicillin or amoxicillin against *C. trachomatis,* it does offer advantages, as already stated, over these other penicillins. However, ampicillin or amoxicillin could be used for the treatment of *C. trachomatis* cervicitis in pregnancy. In the non-pregnant patient, Augmentin would be preferable to the other oral penicillins because of its broader spectrum of activity, thus playing a greater preventive role with regard to fallopian tube damage. In this instance, the additional cost of Augmentin may be justified. However, further investigation of the role of this agent in treating female pelvic infection is warranted.

Augmentin, in the present study, proved to be 100% effective in eradicating *C. trachomatis* from the cervix. One patient was initially treated and found to be cured, but was subsequently found to be positive, re-treated, and cured. Thus, Augmentin appears to be a suitable agent for the treatment of chlamydial cervicitis in the pregnant and non-pregnant patient.

### REFERENCES

1. Centers for Disease Control: *Chlamydia trachomatis* infections: Policy guidelines for prevention and control. MMWR 34(Suppl 3s):53s–74s, 1985.
2. Thompson SE, Washington AE: Epidemiology of sexually transmitted *Chlamydia trachomatis* infections. Epidemiol Rev 5:96–123, 1983.
3. Martin DH, Pastorek JG, Faro S: Risk factors for *Chlamydia trachomatis* in a high-risk population of pregnant women. In Oriel D, Ridgway G, Schachter J, et al. (eds): Chlamydial Infections. Cambridge: Cambridge University Press, p 189, 1986.
4. Martin DH, Koutsky L, Eschenbach DA, et al.: Prematurity and perinatal mortality in pregnancies complicated by maternal *Chlamydia trachomatis* infections. JAMA 247: 1585–1589, 1982.
5. Wolner-Hanssen P, Paavonen J, Kiviat N, et al.: Ambulatory treatment of suspected pelvic inflammatory disease with Augmentin, with or without doxycycline. Am J Obstet Gynecol 158(3 pt 1):577–579, 1988.
6. Centers for Disease Control: Sexually transmitted disease treatment and guidelines, 1985. MMWR 34(Suppl 4s): 75s–108s, 1985.
7. Bowie WR, Alexander ER, Holmes KK: Eradication of *Chlamydia trachomatis* from the urethras of men with nongonococcal urethritis by treatment with amoxicillin. Sex Transm Dis 8:79–81, 1981.
8. Csango PA, Gundersen T, Martinsen IM: Effect of amoxicillin on simultaneous *Chlamydia trachomatis* in men with gonococcal urethritis: Comparison of three dosage regimens. Sex Transm Dis 12:93–96, 1985.
9. Platt MS: Neonatal *Haemophilus vaginalis* infection. Clin Pediatr 10:513, 1971.
10. Sanders LL, Jr, Harrison HR, Washington AE: Treatment of sexually transmitted chlamydial infections. JAMA 255:1750–1756, 1986.
11. Toomey KE, Barnes RC: Treatment of *Chlamydia trachomatis* genital infection. Rev Infect Dis 12(Suppl 6): s645–s655, 1990.
12. Pearlman MD, Faro S, Riddle GD, Tortolero G: In vitro synergy of clindamycin and aminoglycosides against *Chlamydia trachomatis*. Antimicrob Agents Chemother 34: 1399–1402, 1990.
13. Martens MG, Faro S, Maccato M, Riddle G, Hammill H, Wang Y: In vitro susceptibility testing of clinical isolates of *Chlamydia trachomatis*. Infect Dis Obstet Gynecol 1:40–45, 1993.
14. Martin DH, Pastorek JG, Faro S: In vitro and in vivo activity of parenterally administered β-lactam antibiotics against *Chlamydia trachomatis*. Sex Trans Dis 13:81–87, 1981.
15. Bowie WR: In vitro activity of the clavulanic acid, amoxicillin, and ticarcillin against *Chlamydia trachomatis*. Antimicrob Agents Chemother 29:713–715, 1986.
16. Martens MG, Faro S: β-lactam antibiotics and *Chlamydia trachomatis*. Adv Ther 5:113–118, 1988.
17. Kirshon B, Faro S, Phillips LE, Pruett K: Correlation of ultrasonography and bacteriology of the endocervix and posterior cul-de-sac of patients with severe pelvic inflammatory disease. Sex Transm Dis 15:103–107, 1988.
